# Examining the place of the female condom in india’s family planning program aqualitative investigation of the attitudes, opinions of key stakeholders in Pune, India

**DOI:** 10.1186/s12889-022-14054-3

**Published:** 2022-09-05

**Authors:** Medhavi Weerasinghe, Shubhangi Agawane, Neelima Karandikar, Jane Fisher, Jayagowri Sastry

**Affiliations:** 1 Division of Planetary Health, School of Public Health and Preventive Medicine, Monash University, 553 St Kilda Rd, Melbourne Victoria 3004 , Melbourne, Australia; 2 Department of Community Medicine, Smt. Kashibai Navale Medical College and General Hospital, 49/1, Off Mumbai Pune Bypass Rd, Narhe, Pune, Maharashtra 411041, Mumbai, India; 3 Community Researcher, Pune, India

**Keywords:** Female condoms, Contraception, Family planning, Reproductive health, Women-initiated contraception

## Abstract

**Background:**

With overpopulation contributing to the depletion of planetary resources, the high rates of unintended pregnancies in India are a cause for concern. Despite the free supply of contraception options within India’s national family planning initiatives, women are generally offered hormonal options as temporary spacing methods. However, female condoms, a much neglected but potent woman initiated, non-hormonal multipurpose prevention device, are yet to be considered for inclusion in India’s contraceptive cafeteria. Thus, we aim to examine the place of female condoms among the contraceptive options, by analysing the perceptions of key stakeholders regarding its advantages and disadvantages, along with their opinions on how female condoms should be promoted.

**Methods:**

We used purposive sampling to recruit and interview potential users and dispensers of the female condom. The interview participants visited or worked at family planning clinics in Pune at Smt. Kashibai Navale Medical College and General Hospital (SKNMC-GH), its urban and rural outreach clinics, and at Saheli (a non-governmental organisation for female sex workers). We conducted semi-structured interviews and coded our data inductively.

**Results:**

We interviewed 5 rural women, 20 urban women (including 10 female sex workers), 5 male partners of female sex workers, and 5 family planning healthcare providers. Nearly half (12/25) of the women we interviewed, said that they were eager to use female condoms in the future. Many participants perceived female condoms to be an instrument to empower women to be in control of their sexual and reproductive lives (15/35), and that it provided user comfort and confidence (4/35). Their perceived disadvantages are that they are relatively more expensive (6/35), users have limited experience (9/35), and women who buy or use them may be stigmatised and feel embarrassed (4/35). Yet, nearly three-quarters of potential users (21/30) and most healthcare providers (4/5), were confident that female condoms could become popular following extensive promotional campaigns, interventions to improve availability and access, and initiatives to enhance the knowledge of female users.

**Conclusions:**

Female condoms have garnered support from both users and dispensers and have the potential to be widely adopted in India if family planning initiatives which increase awareness, knowledge, and access are systematically undertaken as with other contraceptive options.

## Plain English summary

Female condoms, a woman-initiated and non-hormonal contraceptive, which protects against sexually transmitted infections and unintended pregnancies, are an important but neglected spacing device. Therefore, we aim to examine the role female condoms can play within family planning initiatives of a developing country with high levels of unintended pregnancies. We did this by interviewing key stakeholders in India’s Family Planning Program to seek their current perceptions on the advantages and disadvantages of female condoms, along with their advice on how female condoms should be promoted.

We selected 25 sexually active women from rural and urban areas including sex workers, 5 male partners of female sex workers, and 5 family planning healthcare providers from 4 locations across Pune, a city in West India, to interview in depth. We based our analysis on themes extracted from these interviews.

According to the participants, the perceived advantages of female condoms were that they would empower women to take control of their own sexual and reproductive health, they have no adverse side-affects, and may provide women with confidence to prevent pregnancies. The perceived disadvantages of female condoms were that they are relatively expensive, users have limited experience, and women who buy or use them may be stigmatised or feel embarrassed. However, most respondents said female condoms could become popular following extensive promotional campaigns, interventions to improve availability and affordability, and initiatives to enhance knowledge of female users.

Our study shows that female condoms are largely supported by users and dispensers and have the potential to be widely adopted in countries such as India, if family planning initiatives endorse them by increasing awareness, access, and knowledge.

## Background 

Overpopulation is one of the biggest challenges that our planet faces today, particularly in low-and-middle-income countries (LMICs). For instance, in countries such as India where overpopulation has been a pressing concern since 1952, a “two-child family” is the norm advocated by the government [1]. This has also been accompanied by goals to delay age at marriage, postpone first pregnancies, and space the second one [2]. If these spacing goals are to be achieved, safe and effective temporary methods of contraception are required.

While viewing the various methods available for contraception through a gendered lens, it is obvious that by virtue of having to go through a pregnancy or an abortion, women are disproportionately affected. This would require the following considerations: that in most LMICs it is often women who are expected to employ contraceptives to achieve national population goals; that most temporary methods of contraception (e.g., intrauterine devices, contraceptive pills, and injections) require regular and timely action by women, are hormonal and often invasive to wear with mild to severe side effects; the fact that the only well-known temporary contraception that is a non-invasive and non-hormonal multipurpose prevention technology (MPT) which protects against unwanted pregnancy and sexually transmissible infections (STIs) is the male condom, which requires men to act at the time of coitus. Male condoms can prevent unwanted pregnancies 98% of the time if used correctly and consistently, and 87% if not used properly [3]. Surprisingly then, a 2019 United Nations study on contraceptive use found that male condom usage levels in LMICs was 4.4.% [4]. Then consider the striking point that we do in fact have its less-known counterpart, a female initiated MPT, non-hormonal, non-invasive female condom, which also protects against unwanted pregnancies 95% of the time when used correctly and consistently, and 79% of the time during common use [3]. Furthermore, the female condom can be inserted up to 8 h prior to sexual intercourse, is not dependent on the male erection, and does not require immediate withdrawal following ejaculation [5, 6]. It is inexplicable that there have not been more efforts directed into making female condoms available as an option for women in the same way as male condoms, even in settings such as India where overpopulation, patriarchy, and unintended pregnancies are huge challenges [2, 7–10].

Since as early as 1967, India’s family planning programme provided a cafeteria approach with a ‘basket of choices’ containing a mix of contraceptive methods [11]. Since then, the ‘basket of choices’ has expanded to include 5 official methods — female sterilisation, male sterilisation, intrauterine contraceptive device (IUD), oral contraceptives, and male condoms [12]. Currently male condoms are the cheapest form of reversible contraception in India. A pack of 10 male condoms costs Rs 180 (approximately $2.40USD), while a pack of 2 female condoms are sold for around Rs 100 (approximately $1.30USD) [13]. This difference in price is because female condoms are made from synthetic latex or polyurethane and come with a ring, which is more expensive to make compared to a male condom which only requires latex [13]. Moreover, the Indian government, through its public sector hospitals and clinics, is the main provider of contraceptives in the nation, especially free male condoms as a form of social marketing [14]. One company in India has been manufacturing Nirodh, the free male condom that is widely distributed under India’s Family Planning Program, since 1968 [15]. Yet, a study in 2015 found that around half of the 48.1 million pregnancies in India were unintended, and that 15.6 million pregnancies ended in abortions [16]. India’s National Family Health Survey (NFHS) found that male condom usage rates are estimated to be only 10.2% in the state of Maharashtra, where this study was undertaken, with 14.1% in urban cities and 7.1% in rural towns [17]. Reported barriers that impede the usage of male condoms in India include gendered sociocultural expectations, lack of knowledge, and unevenly skewed contraception decision making power in favour of the male partner [18–21]. Moreover, male condoms must be negotiated between partners, leaving women with minimal bargaining power if their male partner refuses to wear it [22].

Though female condoms have the potential to spearhead an era in India’s family planning history where women can take full control over their own sexual health, they have continued to be overlooked as a viable and important addition to India’s contraceptive cafeteria. Along with other less popular modern contraception methods (e.g., the diaphragm), female condoms are one of the contraceptives utilised by less than 0.5% of users in low-and-middle-income countries [4]. Despite the existence of studies which assess acceptably and promote women’s agency and other numerous positive attributes of female condoms in countries such as India [23–25], it appears that female condoms are still widely unknown, and have not been placed within, let alone at the forefront of national contraceptive programs [4, 26, 27]

Consequently, there appears to remain a more or less ethical necessity to re-focus attention on the multipotent but much-neglected female condoms, and to activate the debate on a larger role that they could potentially play as a contraception option in LMICs. Thus, as a first vital step, we aimed to investigate the perceptions of potential users and dispensers of female condoms in the following three domains: (1) perceived advantages of female condoms, (2) perceived disadvantages of female condoms and, (3) recommendations for future promotions of female condoms in India’s Family Planning Program.

## Methods

### Setting and study participants

Between May 2015 and September 2016, we undertook a multi-centric qualitative study at four sites in Pune, a city in West India. Pune is India’s 9th largest city, with a population of over 5 million, residing in both urban and rural regions of the state. Furthermore, the Budhwar Peth red-light area in the Pune city is home to one of the largest brothel-based sex industries in India.

We tried to account for Pune’s diverse demography by using purposive and convenience sampling to identify 35 interview participants who lived in urban and rural areas of Pune and were both at high and low risk of unintended pregnancy and STIs, as well as family planning healthcare workers who dispense contraceptives. Thus, we conducted interviews with female sex workers from Saheli, a non-governmental organisation for sex workers, some of whom had heard about or used female condoms in the past, and their male partners, all of whom had used female condoms in the past. As well as inviting the participation of women, who had never previously heard about or used female condoms, from the family planning clinic in Pune at Smt. Kashibai Navale Medical College and General Hospital (SKNMC-GH), an urban tertiary care hospital, and its urban and rural outreach centres. We also interviewed healthcare providers from all four sites.

### Procedure

Guides for the qualitative interviews of participants were developed by JS and social scientists in Pune who were fluent in Marathi, Hindi, and English. While the interview guides focused on the knowledge and experiences participants had of dispensing/using various contraceptive methods, in this paper, we focused particularly on their views regarding the advantages and disadvantages of female condoms, and their opinions for future promotions of female condoms in India’s Family Planning Program.

Our research team consisted of 6 trained social scientists, who collected our interview data, and JS. Everyone was trained on the protocol, the anatomy of the female genital tract, the prevalent methods of contraception at the study sites, and contributed to the development and translation of our interviews. Additionally, they were provided with detailed information on the female condom, including practice inserting it into a mannequin.

All interviews were face-to-face and conducted privately either at the participant’s home, at the clinic they were attending, or any other location of their choice. We conducted semi-structured interviews to allow participants to provide spontaneous responses to inform our research without being restricted by rigid questions as observed by Leech [28]. To avoid misinterpretations, two interviewers attended each interview, one of whom took field notes, while the other conducted the interview and recorded responses on a tape-recorder (participants provided written consent to have their interviews recorded). We conducted the interviews in Marathi or Hindi, according to the participant’s preference, and translated them to English during the transcription process. To ensure validity and accuracy during the translation process, translators referred to both the tape recording and field-notes. Moreover, the original interviewers read over and double-checked the translated scripts. To ensure impartiality during the coding process, all script were de-identified.

### Data management and analysis

All study forms and interview data were anonymised and stored on a secure, password protected server in the Clinical R&D department at Smt. Kashibai Navale Medical College and Hospital, that could only be accessed by members of the study team. We used an inductive coding approach to form themes based on our interview findings [29]. Our analysis team members read the first interviews of one participant from each category very carefully, and several times for familiarity, and to write up a summary of each interview. We then created a preliminary codebook based on the open coding we performed on the first interviews. Throughout the remaining data collection and analysis process, we met once a week to revise the initial coding list, resolve any discrepancies between codes, and create additional codes if necessary. We then imported the interview transcripts into NVivo 12, a software designed to generate thematic nodes and conduct content analysis on qualitative data. The nodes generated through NVivo were incorporated to the themes we found through open coding to substantiate and finalise our codebook. We further categorised and organised the codes with relevant quotes extracted from all the interviews and conducted the final analysis. Manuscript drafts were shared with the research team in India, to enable us to include their comments.

### Ethics and informed consent

Our study was approved by the Institutional Ethics Committee at Smt. Kashibai Navale Medical College and Hospital, Pune. All interview participants were over the age of 18, and we obtained written informed consent from them to discuss in detail their opinions on female condoms. Furthermore, we provided them with Rs 150 (approximately $2.25USD) as compensation to cover the cost of transportation to attend the interviews.

## Results

We talked to 35 key stakeholders from rural and urban Pune about the opportunities and challenges for including female condoms as a contraceptive option for women in India’s family planning contraceptive cafeteria. We interviewed 15 sexually active women, 5 from the SKNMC-GH main hospital, 5 from its urban Pune outreach clinic, and 5 from its rural Pune outreach clinic. From Saheli we interviewed 10 female sex workers, and 5 male partners of female sex workers. Additionally, we carried out interviews with key informants including 5 family planning healthcare providers (2 Nurses, 2 Counsellors, and 1 Gynaecologist) who worked at one of these four sites. The interviews lasted for a duration of 45 min to an hour, and each interview was attended by the participant and two interviewers. To ensure that all respondents could express their opinions on female condoms, which are less known in India, interviewers explained and demonstrated how female condoms are used before starting the interview. The number of interviews conducted, and characteristics of the interview participants are shown in ‘Table 1’. The findings from our interviews are categorised into the following themes:

**Table 1:** Characteristics of Interview Participants

	Participant Category	Site	No.	Mean age (years)	Education				Marital Status			Knowledge / Used or Dispensed Female Condom Prior To Study
					Non-literate	Primary	Secondary	Graduate	Single	Married	Separated	
**1**	**Women (Potential Users)**	SKNMC-GH (Urban Participants)	5	28	0	0	3	2	0	5	0	Nil / Nil
		SKNMC-GH Urban Health Training Centre (Urban Outreach Participants)	5	26.2	0	0	4	1	0	5	0	Nil / Nil
		SKNMC- Rural Health Training Centre (Rural Outreach Participants)	5	23.8	0	0	4	1	0	5	0	Nil / Nil
		Saheli NGO (Female Sex Workers)	10	26.8	6	2	2	0	3	6	1	All / 4
**2**	**Men Users** **(Potential Users)**	Saheli NGO (Male Partners of Female Sex Workers)	5	36.4	0	1	4	0	2	3	0	All / All
**3**	**Healthcare Providers: Doctors/ Nurses/ Counsellors**	Across all four sites (4 rural, 1 urban)	5		0	0	0	5				All / 1
**Total**			35									

### Perceived advantages of female condoms by stakeholders

Once the participants had seen and understood how female condoms are used, almost half of the women we interviewed – 3/5 rural and 9/20 urban—conveyed their eagerness to use it in the future. One urban woman from the main hospital (a graduate) stated that: “… It is good, it is meant for our safety. Women will definitely use it because they have a great fear of pregnancy. Women should not have this fear in their mind.”

#### Women-initiated dual protection contraceptive

More than a third of potential users [15], including 5 urban women, 2 rural women, and 4 Saheli participants, appreciated the fact that female condoms could provide leverage for women to take full control over the entire contraception process. For example, one urban woman (a graduate) said that: “It is a good thing that women can have their own protection today. There is no need to depend on others.” Similarly, a rural woman (who had completed her secondary education) mentioned that: “…If our husband has sexual relations with someone outside, we may get STDs… To avoid this, we women should use female condoms to protect ourselves.” Another urban woman (with secondary education) explicitly linked this control to her capacity to grow as a person: “…If you had met me before, the fears I had in my mind would have gone away, and I could have used it [female condom] to prevent an early pregnancy. I would have conceived at the time I wanted and hence I would have my own personal growth…” Although she had not previously used a female condom herself, a female sex worker from Saheli (who was not literate) detailed how female condoms had been used among her colleagues to protect themselves whenever male customers refused to wear male condoms: “…They would go to the toilet and come out wearing a female condom. The customer would not be aware because they turned off the lights while they had sex… When the customers drank alcohol, they would also use female condoms.”

#### User comfort and confidence

Four urban women (including 3 female sex workers who had all used female condoms in the past), acknowledged that female condoms had no adverse side-affects and provided women with a peace of mind during sexual intercourse. It was expected that respondents from Saheli would be more vocal about the benefits of female condoms as they had some experience using it as opposed to other women participants. For example, a female sex worker from Saheli (with secondary education) explained that: “Sometimes when we use government male condoms, they get torn and remain stuck inside us. But female condoms are fixed when inserted inside. Afterwards we can go to the toilet and take it out. It does not get torn and nothing bad happens…” However, one woman from the urban outreach clinic (who had a postgraduate qualification), also commented that if female condoms were more available, she would no longer have to risk her health by using copper IUDs and consuming contraceptive pills, which are the only female initiated reversible contraceptives available at family planning clinics: “…We have to consume pills even though we have no sexual contact (intercourse)… The Copper-T is also continuously fixed in our body. This [female condom] is not like that right? We only have to use it when we are having intercourse. That is why it is better.”

### Perceived disadvantages of female condoms by stakeholders

#### Stigma and embarrassment

Among the respondents, it was the rural women (2/5) and men from Saheli (2/5) who were sceptical about using female condoms due to stigma and embarrassment. Some women were scared that their regular male partners would become suspicious as to why they want to wear female condoms if they were not using male condoms previously. For example, when talking about the stigma attached to female condoms, a woman from the Rural Health Training Centre (with secondary education) professed that: “…Every man has some doubts and suspicions in his mind…I have heard about how they suspect their wives of having extra martial relations… In a way, women have some sorts of bindings on them. If he is educated, he can understand why his wife wants to use a female condom, but the use of female condoms might be dangerous when the husband is illiterate.” She linked these assumptions of infidelity to feelings of embarrassment: “Men, including my husband, will not like their wives going to a medical store and asking for [female] condoms in front of male customers. Women themselves would feel embarrassed to buy it…”.

#### Physical barriers

Nearly a quarter of the respondents [9], mostly urban women (4/6 were female sex workers whom we interviewed at Saheli), who had no prior experience inserting and using female condoms during sexual intercourse, voiced their concern about its size. One female sex worker (who was not literate) stated: “I think that a female condom looks too big, so I am a bit worried about how to use it…”.

Participants also reported that reluctance stemmed from the fact that female condom is difficult to obtain due to its high prices. If women cannot negotiate the use of male condoms, which are provided for free by the Indian government or are cheap to purchase, the likeliness that they will turn to female condoms as an alternative is low due to its comparatively higher price. A nurse we interviewed explained this to us: “…Female condoms are a little costly. Even though we make some contraceptives free, people do not take advantage of it. So, even if we tell them to buy it, if it is a little costly, they will not choose it…”.

### Stakeholder recommendations for future promotions of female condoms

Most participants (25/35) were confident that following targeted interventions, female condoms would be accepted by users within the contraceptive cafeteria in India. Although only 2/5 rural women supported this view, almost all healthcare providers (4/5), urban women (9/10), and participants from Saheli (7/10 female sex workers and 3/5 male partners), were of this opinion. One Nurse emphasised that: “Whenever our society is introduced to something new the initial reaction is ‘no’. However, after knowing its advantages, people automatically start using it.”

#### Extensive promotional campaigns

Participants expressed that the first step to popularising female condoms as a contraceptive option is to ensure that the public are made aware of its existence and all its merits as one of the few reversible dual-protection contraceptives which women have full control over. One male partner from Saheli (who had completed his secondary education) urged that: “You will have to stick pamphlets on buildings, in public bathrooms, on railway stations, and our local bus stops etc. Wherever there is public movement you should stick advertisements so that the public will read them…They will come to know where female condoms are available and will go and buy it.”

Nearly half of the urban users and dispensers (13/29) emphasised that televisions and radios are the most effective mediums through which female condoms should be promoted. All the participants who mentioned broadcasting media were residing in urban Pune where they are frequently exposed to electronic mass-communication channels and are more likely to be in possession of such devices as opposed to individuals living in rural villages. For example, one male user from Saheli (with primary education) stated that: “Advertisements for female condoms should be played on the television and radio every hour or every half-an-hour between programs…These advertisements will reach every house…”.

#### Improved access

Respondents also advised that it was essential to make female condoms more accessible, both in terms of availability and price. Most of the male partners from Saheli (4/5), healthcare providers (3/5), as well as a few women participants (1 rural, 2 urbans, and 1 sex worker), stressed that levels of public acceptance would not increase unless campaigning efforts were matched with an upsurge of female condom supplies. For example, a male partner from Saheli (with secondary education) said that: “…Female condoms should be made available at every medical store and general store. If you start supplying female condoms to these shops, then everyone will know what it is and where it can be brought…” Furthermore, there was a consensus that the benefit of female condoms would only be truly appreciated if they could be accessed conveniently, and in women-friendly spaces: “It should be available in different stalls, small shops or medical shops… At Rural Health Centres… In vending machines where women can insert coins to get female condoms” (Counsellor), and can be brought by women in confidence: “There are shops for women such as beauty parlours… Places where only women go to shop… so that women do not feel embarrassed about who is beside them, such as if there is a man… If female condoms are available at such places, then women will purchase it” (Woman, urban outreach, with a graduate qualification).

Moreover, 6 participants (primarily healthcare providers (3/5)), stated that female condoms should be offered for free or sold at a low price so that everyone could afford it. For example, a family planning counsellor explained that: “…Women need to get female condoms free of cost. She needs to be able to easily get it first and then she will become ready to use it…” Most family planning clinics in India only offer copper IUDs and contraceptive pills to women. This was common practice according to women who attended family planning clinics. For instance, one woman from the main hospital (with secondary education) said: “Our doctor told us that our daughter is very small, don’t take tablets, use the Copper-T it is best for you…He did not tell us about other methods…” A Gynaecologist working at the urban family planning clinic also said: “…We have only two contraceptive options. If their children are big, women undergo operation. If their child is small, the Copper-T is inserted.” However, to enable access, an urban women (a graduate) voiced her desire to have female condoms as a third free option at these clinics: “…If it is available for free at the entrance of women’s departments in hospitals, where women generally visit…Then women will surely use it at least once. After they first try it, they will become aware of its effectiveness and will use it forever…” This was also deemed important by a woman living in rural Pune (who had completed her secondary education): “If they are kept in dispensaries and hospitals with lady (female) doctors… then it is good for women because they do not have to buy female condoms openly in the market.”

#### Knowledge of female users

More than a third of the participants, including most healthcare providers (4/5) and some women from Saheli [3], stressed the significance of increased knowledge and awareness, which would preclude women from being afraid to buy and use female condoms: “Women should be given information about this… Women should be told how female condoms are to be used, because after buying it directly from the medical store, they don’t know how to use it…” (Woman, urban outreach, a graduate). Additionally, almost two-thirds of the rural participants (3/5) and half of the urban women (7/15), advocated for the organisation of community meetings where women can come together to collectively learn about female condoms, support each other when practising how to use female condoms, and have a forum to freely discuss their views on female condoms. One rural woman (with secondary education) was impassioned enough to state that: “…Meetings should be arranged in hospitals or in schools. All women should gather there, and it should be explained to them that female condoms are for their safety. Do you know what life is like for a woman here [rural region of Pune]? They are confined to the kitchen and children…For those women, meetings should be organised, and they should be given information about female condoms…” The potency of ‘women only’ meetings is that women do not feel judged or guilty when speaking openly about their experiences using female condoms: “…For women it is better to keep some occasion for meeting rather than meeting at their home… When you [health professionals] go to their house there may be guests, children, or their mother-in-law, so we cannot talk in front of them. Even if the woman wants to speak about female condoms, she may not be able to…” (Woman, main hospital, with a graduate qualification). A female sex worker from Saheli (who was not literate) also mentioned the importance of organising these gatherings to ensure that all women are introduced to female condoms: “Brothel owners should be called for special meetings about female condom use…The girls [sex workers] will also come with them to listen…”.

## Discussion

### Discussion of results

Our study is one of the first to look at the place for female condoms in India’s Family Planning Program based on the views of a wide range of users/potential users and dispensers (high risk and low risk), from urban and rural villages in India. Our study also included the experiential views of female sex workers and their male partners who had a history of female condom use. In general, we found that participants viewed female condoms as a welcome addition to their current basket of contraceptive choices and believed that it could become popular among users and dispensers following targeted interventions. We discuss our results below according to the World Health Organisation’s ‘Acceptability, Availability (and affordability), Accessibility, and Quality’ (AAAQ) framework, which considers access to health care from a human rights perspective in settings such as India, while taking quality for granted in this study [30].

#### Acceptability

When questioned about the advantages of female condoms, the views of those we interviews aligned to a degree with the findings of Weeks et al. (2013), who found that female condoms are advantageous to women (particularly female sex workers) as they can use it with their regular, casual, and paying partners [31]. Moreover, similarly to our findings, it has also been identified that female condoms are desirable as they provide protection for women without negatively affecting their hormones or having harmful side-effects [32]. This is important because intrusive contraceptive devices may take a toll on women. For example, in India, 25.5% of women who used modern non-permanent contraceptives report discontinuation due to non-pregnancy/fertility related factors [33].

On the other hand, some of the perceived barriers voiced by participants are perhaps rooted in misconceptions due to inadequate exposure to female condoms. For instance, a concern expressed by 4 respondents was that female condoms are a symbol of infidelity. A study conducted by Bandewar et al. (2015) in India also found that if women insisted on using male condoms they would be questioned on their commitment to their regular partner as “love was seen as incompatible with condom use” [21]. However, our study interestingly also found that this was a reservation held only by women interviewed at rural sites and not women interviewed at urban sites. This could be explained by the ‘diffusion of innovation’ theory, according to which rural populations are usually a part of the ‘late majority’ who are hesitant to adopt novel innovations compared to their urban counterparts who make up the ‘early majority’ [34]. Hence, if many urban users and dispensers start utilising female condoms, it could lead to a city-to-rural diffusion of female condom uptake [34]. Future investigations into the promotion of female condoms according to the ‘diffusion of innovation theory’ would be useful. Moreover, male condoms have come to be associated with infidelity because of the extensive community campaigns advocating their use to prevent transmission of the human immunodeficiency virus (HIV) [21]. For the time being in India, female condoms are in a sense, ‘protected’ from this ‘notoriety’ as they are still widely unknown, and as a result, have the chance to be marketed to the public more as contraceptives that can prevent STIs, than vice-versa.

We have attempted to contribute novel insights to the wider scholarly discussion surrounding how to dismantle barriers and increase the uptake of female condoms by users. Based on the views of our interview respondents, we found that wider visibility of female condoms by making them available in public clinics and through various media might help familiarise women with the device and reduce user apprehensions [35]. Additionally, family planning agencies in India should develop creative promotional materials in different mediums which showcase the full potential of the female condom, as a multipurpose prevention technology. As it was the urban users and dispensers who largely believed that female condoms would be accepted within India’s family planning contraceptive cafeteria, promotional and educational efforts should also be initially focussed on the urban districts of India, and then extended into rural villages for greater acceptance.

#### Affordability and availability

Another perceived disadvantage mentioned by participants was that the higher cost of female condoms compared to male condoms, coupled with its limited availability, makes it inaccessible in resource constrained settings such as India. This was also a re-emerging theme presented by other researchers from Zambia and South Africa [6, 36, 37]. Thus, although female condoms have been introduced in many countries, their supply and uptake in developing nations hardest hit by HIV, and with high rates of abortions is largely inadequate [38].

The ease of use, and consistent use can happen only if availability is perineal, and practice makes perfect [27]. In our study, we found that while potential users and dispensers are willing and enthusiastic to make use of female condoms, the continued underutilisation of this vital female initiated dual-barrier contraceptive can be largely accounted for by a shortage of targeted publicity, availability, and educational programs, because they are not an option in India’s family planning cafeteria. In fact, Peters et al. (2010) critique the fact that female condoms have never been in the limelight since they were introduced in 1984, and their analysis revealed that the strong international potential of female condoms has been stymied mainly at the international policy levels rather than any obstacles from the users end [27]. Compared to male condoms that have been widely promoted, female condoms are relatively unknown even though, like male condoms, India has also been domestically manufacturing and distributing female condoms since 2012. In fact, the country has even introduced a more affordable variation of the female condom made from natural rubber latex [39, 40]. Thus, there should be no dearth in availability if India’s Family Planning Program were to offer it as an option as many participants in our study favoured the free provision of female condoms. Additionally, it is expected that widespread use may further drive down their costs. Hence, although female condoms are currently more expensive than male condoms, the vicious cost-availability-supply circle can best be broken through social-marketing where like male condoms, female condoms are offered for free through India’s public family planning initiatives.

While the recommendations of increasing stock and reducing prices have been commonly cited in other studies [41, 42], we found that a marketing strategy to promote the use of female condoms in India should also address concerns linked to infidelity and embarrassment by assuring women-friendly spaces and anonymity. This would enable women to confidentially purchase female condoms. A more optimal and non-stigmatising way would be to offer female condoms routinely as an option in family planning clinics at various health centres that women visit, along with all other conventionally offered contraceptives.

#### Accessibility

Like participants in two other studies in India and Zambia [6, 35], a few of the women we interviewed were also hesitant to use female condoms, as they feared inserting it into their vagina. We found this to be largely articulated by women who had no prior experience using female condoms. Thus, to improve user knowledge and confidence, the features of female condoms which enable user comfort, such as pre-lubrication, and flexibility of the rings at each end of the female condom, need to be emphasised while counselling women for its use. Additionally, to dispel fears that arise due to unfamiliarity, there should be detailed demonstrations of how female condoms are inserted, and women should be offered the option to practice insertion on mannequins. Furthermore, based on the grassroot recommendation made by respondents, we found that community organised women-friendly meetings are more effective avenues to disseminate information on female condoms as opposed to top-down initiatives. Consequently, family planning agencies should collaborate with trusted grassroot clinics, NGOs, and female community leaders to organise local gatherings where women can discuss the benefits of female condoms amongst themselves and support each other when learning about how to use it.

### Limitations of method

Our study utilised purposive sampling for identifying interview participants at 4 locations in the city of Pune, West India. Pune was chosen as the site for this study, as it was in a unique position of providing us with the views of some women and their partners who had lived experiences of using female condoms. Considering that this is the first study on users’ perception of a potential role for female condoms in the contraceptive cafeteria, it is expected that findings from this study would provide the basis for building further focussed qualitative and quantitative studies in other regions of India, particularly in the Empowered Action Group (EAG) states to guide further policies family planning in India. As participants were predominantly urban residents [29], the views of rural stakeholders are limited to 6 participants (5 women and 1 nurse). Thus, the perspectives of these respondents may not directly reflect views of all relevant stakeholders residing in other regions of India.

Moreover, this study was qualitative in nature, with 35 participants, and while it provides nuanced details into the experiences and views of key stakeholders, the findings may not be generalisable and should be supplemented with larger mixed method studies in different regions of India. To bring about a change in the policy to include female condoms as an option in the national Family Planning program, further qualitative research that includes other stakeholders such as women’s husbands, mothers-in-laws, and brothel owners, as well as larger quantitative surveys on representative populations in different regions of India are needed.

## Conclusion

Female condoms are one of the few safe and reversible contraceptive devices over which women have full control, giving them critical agency, particularly in patriarchal societies, to take the protection of their sexual and reproductive wellbeing into their own hands, while contributing to population goals in low-income settings as India. Yet, our study is one of the first to investigate the introduction of this versatile and multipotent contraceptive, which has been undervalued and spurned thus far, into India’s Family Planning Program. We found that there were high levels of support from key stakeholders for female condoms as they protect women and give them leverage in ways that other female initiated contraceptives fail to do. Even though participants voiced their concerns about their fear of stigmatisation, embarrassment, and barriers to access, none of these perceived weaknesses are due to the intrinsic nature of the female condom, but are rather the results of contextual factors, which can be resolved through targeted interventions and initiatives. This important point was accentuated through the recommendations pertaining to promotion, availability, and education offered by participants. All recommendations which can be practically and economically acted upon by India’s Family Planning Program. Hence, we argue that the pernicious underutilisation of female condoms, despite user enthusiasm and support, is because of low to non-existent recognition by family planning programs around the world. Therefore, there is an urgent need for LMICs to situate this dynamic contraceptive in the spotlight and reanimate the debate on the potentially pivotal role it could play in population planning.

## Data Availability

The datasets used and/or analysed during the current study are available from the corresponding author on reasonable request.

## Abbreviations

AAAQ: Acceptability, Availability, Accessibility, and Quality (World Health Organisation)
HIV: Human immunodeficiency virus
HLL: Hindustan Latex Ltd
IUD: Intrauterine device
LMICs: Low-and-middle-income countries
MPT: Multipurpose prevention technology
SKNMC-GH: Smt. Kashibai Navale Medical College and General Hospital
STI: Sexually transmissible infection
NFHS: The National Family Health Survey (India)
